# The ANC-1 (Nesprin-1/2) organelle-anchoring protein functions through mitochondria to polarize axon growth in response to SLT-1

**DOI:** 10.1371/journal.pgen.1010521

**Published:** 2022-11-21

**Authors:** Nathan C. Fischer, Vladislav Friedman, Miguel A. Martinez-Reyes, Hongyan Hao, Tamjid A. Chowdhury, Daniel A. Starr, Christopher C. Quinn

**Affiliations:** 1 Department of Biological Sciences, University of Wisconsin-Milwaukee; Milwaukee, Wisconsin, United States of America; 2 Department of Molecular and Cellular Biology, University of California, Davis, California, United States of America; Brown University, UNITED STATES

## Abstract

A family of giant KASH proteins, including *C*. *elegans* ANC-1 and mammalian Nesprin-1 and -2, are involved in organelle anchoring and are associated with multiple neurodevelopmental disorders including autism, bipolar disorder, and schizophrenia. However, little is known about how these proteins function in neurons. Moreover, the role of organelle anchoring in axon development is poorly understood. Here, we report that ANC-1 functions with the SLT-1 extracellular guidance cue to polarize ALM axon growth. This role for ANC-1 is specific to its longer ANC-1A and ANC-1C isoforms, suggesting that it is mechanistically distinct from previously described roles for ANC-1. We find that ANC-1 is required for the localization of a cluster of mitochondria to the base of the proximal axon. Furthermore, genetic and pharmacological studies indicate that ANC-1 functions with mitochondria to promote polarization of ALM axon growth. These observations reveal a mechanism whereby ANC-1 functions through mitochondria to polarize axon growth in response to SLT-1.

## Author summary

The brain is comprised of a network of neurons, with each neuron extending a single axon that makes connections with other neurons. Alterations in these axonal connections are thought to cause brain disorders such as autism, bipolar disorder, and schizophrenia. The risk of these disorders is increased by alterations in mitochondria, an organelle within neurons that is needed for the production of energy and signals that promote normal development of axonal connections. Although mitochondria are critical for normal axon development, the mechanisms that mediate the correct localization and function of mitochondria within axons are not well understood. Here, we identify a protein that promotes the correct localization of mitochondria to the base of the axon. Disruption of this protein causes defects in mitochondria localization and causes defects in axon development. Alterations in the human homolog of this protein have been associated with brain disorders, suggesting a possible explanation of how these disorders arise.

## Introduction

A family of giant proteins, including mammalian Nesprin-1 and -2 (encoded by the *SYNE1* and *SYNE2* genes), *C*. *elegans* ANC-1, and *Drosophila* MSP-300 are defined by containing an N-terminal actin-binding domain, a long central domain related to spectrin, and a C-terminal KASH domain that targets the protein to the outer nuclear membrane [1]. Variants in *SYNE1* have been implicated in neurodevelopmental, neuropsychiatric, and neurodegenerative disorders. For example, the *SYNE1* gene has been implicated in autism by large-scale sequencing studies that have identified several autism-associated de novo variants in this gene [2–6]. Likewise, genetic analysis of a consanguineous family identified a homozygous mutation in *SYNE1* that is causative for autism [7]. Moreover, genome wide association studies have identified risk alleles in *SYNE1* that are associated with schizophrenia, bipolar disorder, and major depression [8–10]. In addition, homozygous protein-truncating mutations in *SYNE1* are a common cause of a form of recessive ataxia that can result in cerebellar degeneration, neuromuscular degeneration, and intellectual disability [11–13]. Despite their widespread roles in neurological disorders, the mechanisms of giant Nesprin proteins in neurons remains poorly understood.

The best understood functions of the Nesprin-1 and -2 protein family occur at the outer nuclear membrane, where their luminal KASH domains interact with SUN proteins in the inner nuclear membrane to form a bridge across the nuclear envelope called the LINC (linker of nucleoskeleton and cytoskeleton) complex [1,14–17]. The cytoplasmic domains of giant Nesprins interact with the cytoskeleton to promote either nuclear anchoring or nuclear movement. For example, ANC-1 is required for nuclear anchoring in the hypodermal cells of *C*. *elegans* [16,18]. Likewise, in muscle cells, Nesprin-1 and -2 promote clustering of nuclei at the neuromuscular junction [19]. ANC-1 can also regulate protein homeostasis through the regulation of gene expression [20]. Within neurons, Nesprin-1 and -2 regulate the interkinetic nuclear movements that promote neurogenesis and neuron migration [21,22]. Moreover, in *C*. *elegans* neurons, ANC-1 functions with the SUN protein UNC-84 to regulate axon termination and synaptogenesis [23].

A less well understood aspect of the giant Nesprin protein family are its non-nuclear functions of anchoring the ER and mitochondria, which occur independently of SUN (UNC-84) proteins. For example, mutations in *C*. *elegans anc-1* lead to mislocalized ER and mitochondria that float freely in the hypodermal cells of *C*. *elegans* [18]. However, UNC-84 is not required for anchoring of ER or mitochondria [18]. Similar observations have been made in muscle cells of both *Drosophila* and *C*. *elegans* [16,24,25]. Consistent with these findings, members of the giant Nesprin family have been localized to both the ER and mitochondria [18,26,27]. Despite these initial findings, the non-nuclear functions of the giant Nesprin family remain relatively unexplored. Moreover, the non-nuclear functions have yet to be investigated in neurons.

Mitochondria are involved in several aspects of neuronal development including the establishment of polarity, axon growth, branch formation and growth cone steering [28,29]. However, the mechanisms that regulate mitochondria to promote development are poorly understood. A key aspect to understand this process is to determine how mitochondria are localized to the correct location within the neuron to promote specific neurodevelopmental steps. For example, a cluster of immobile mitochondria have been observed at the base of the proximal axon and this cluster of mitochondria is required to establish neuronal polarity [30,31]. However, the mechanisms that cause mitochondria to localize to the base of the proximal axon are unknown.

Here, we report that ANC-1 functions in the SLT-1 signaling pathway to promote polarization of axon growth in the ALM neuron. We find that ANC-1 promotes localization of mitochondria to the base of the proximal axon and that ANC-1 functions with mitochondria to polarize axon growth. Analysis of isoform-specific mutations indicate that the axon-growth polarizing activity of ANC-1 is mediated by the longer ANC-1A and ANC-1C isoforms. Consistent with these observations, we find that ANC-1A and ANC-1C are enriched in in the ALM and the shorter ANC-1B isoform is not detected in the ALM. By contrast, prior studies have indicated that the role of ANC-1 in the anchoring of the nucleus is mediated by the shorter ANC-1B isoform. Together, these observations support a model where the SLT-1 extracellular cue functions with ANC-1 to polarize axon growth by recruiting mitochondria to the base of the proximal axon.

## Results

### UNC-6 and SLT-1 extracellular guidance cues polarize ALM axon growth

The *C*. *elegans* ALM mechanosensory neuron can serve as a model to study mechanisms that polarize axon outgrowth. The ALM normally extends a single axon from its anterior side. However, loss of polarizing-components result in a multipolar phenotype, where a second process grows from the posterior side of the ALM. For example, loss of the frizzled receptor or the extracellular Wnt ligand creates a multipolar phenotype in the ALM [32,33]. The ALM has also been used to study several other receptors, channels, and intracellular signaling proteins that can polarize axon growth [34–41].

**Fig 1:**
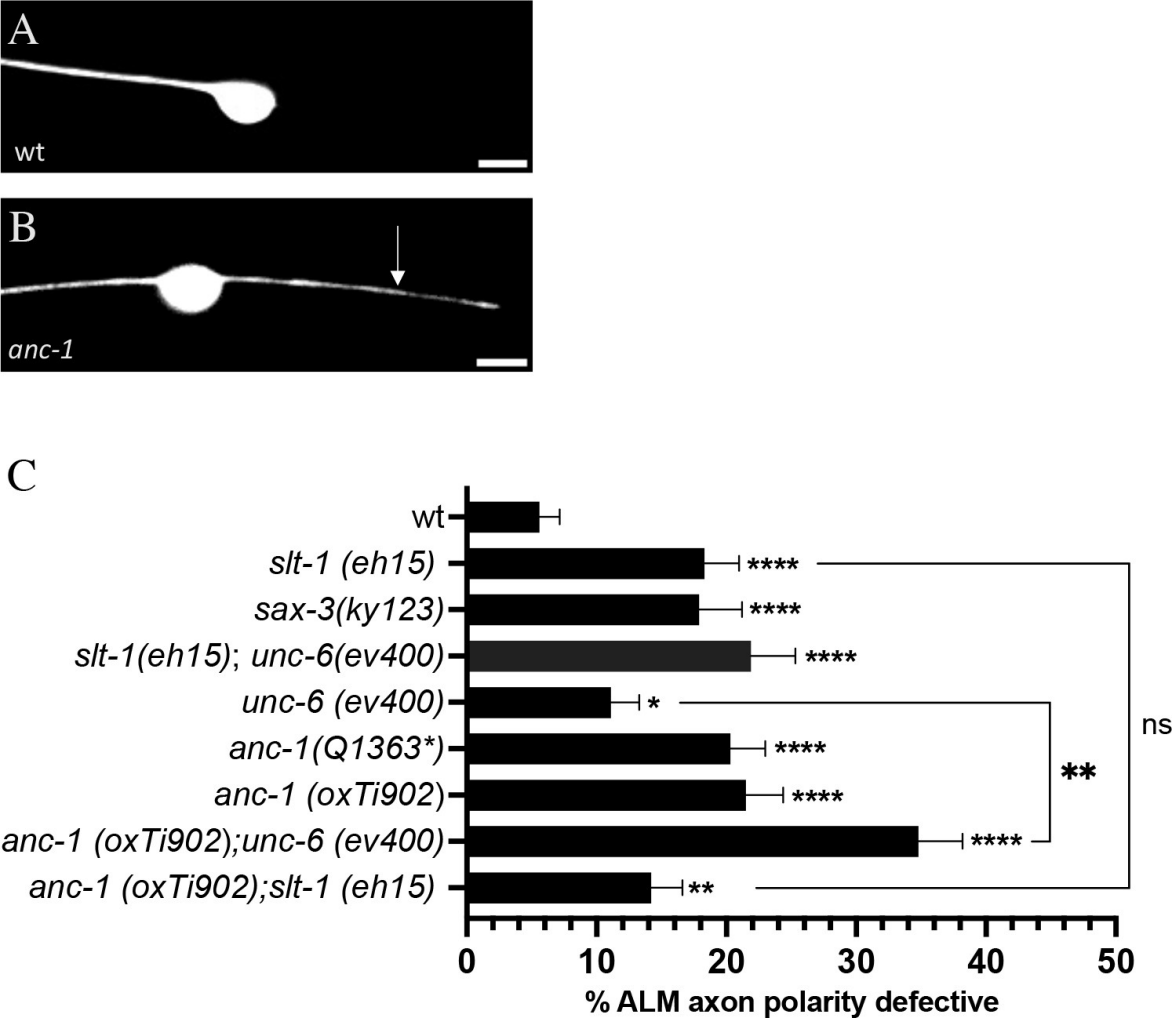
ANC-1 functions with SLT-1 to polarize ALM axon growth. **(A)** Example of a wild-type ALM neuron, with a single process growing from the anterior side of the cell body. **(B)** Example of a multipolar defect in an *anc-1* mutant ALM neuron, with a second process growing from the posterior side of the cell body (arrow). **(C)** Quantification of the ALM axon polarity defects. Analysis of double mutants indicates that *anc-1* and *slt-1* function together in a genetic pathway to polarize ALM axon growth. In addition, *anc-1* functions in parallel to *unc-6* to polarize ALM axon growth. ALM neurons were analyzed in L4 hermaphrodites using the *jsIs973* transgene that encodes *Pmec-7*::*rfp*. N>198 for all genotypes. Asterisks above individual bars indicate statistically significant difference relative to wildtype control, “N-1” Chi-squared test for proportions (*p = .041, **p < .01, ****p < .0001). Asterisks above brackets indicate statistically significant difference relative to wildtype control, “N-1” Chi-squared test for proportions (**p < .01Asterisks). Error bars represent the standard error of the proportion. Anterior is to the left. Scale bars are 5 μm.

To further explore the mechanisms that polarize axon growth, we sought to identify additional extracellular cues that can polarize ALM axon growth. We considered UNC-6 and SLT-1 as candidates because both are known to affect various aspects of neuronal polarity [41–48]. For these experiments we analyzed ALM axon growth in null mutants of *unc-6* and *slt-1*. In wild type, ALM axon growth was almost always polarized, such that a single axon grew from the anterior side of the cell body (Fig 1A and 1C). In *slt-1(eh15)* null mutants, we found that a second process grew from the posterior of ALM with a penetrance of 18% (Fig 1B and 1C). We found a similar phenotype in *unc-6(ev400)* null mutants, albeit at a substantially lower penetrance. To test for a role of SAX-3, the receptor for SLT-1, we used the *sax-3(ky123)* null mutation. We found that *sax-3(ky123)* null mutants exhibited ALM axon polarity defects with a penetrance similar to *slt-1(eh15)* mutants (Fig 1C). To address the relationship between SLT-1 and UNC-6 in the polarization of ALM axon growth, we analyzed *unc-6(ev400); slt-1(eh15)* double mutants. We found that the penetrance of axon polarity defects in *unc-6(ev400); slt-1(eh15)* double mutants was not significantly different than the penetrance of axon polarity defects in *slt-1(eh15)* single mutants (Fig 1C), suggesting that *unc-6* and *slt-1* do not function in parallel to polarize ALM axon growth.

### ANC-1 functions in the SLT-1 signaling pathway to polarize ALM axon growth

**Fig 2:**
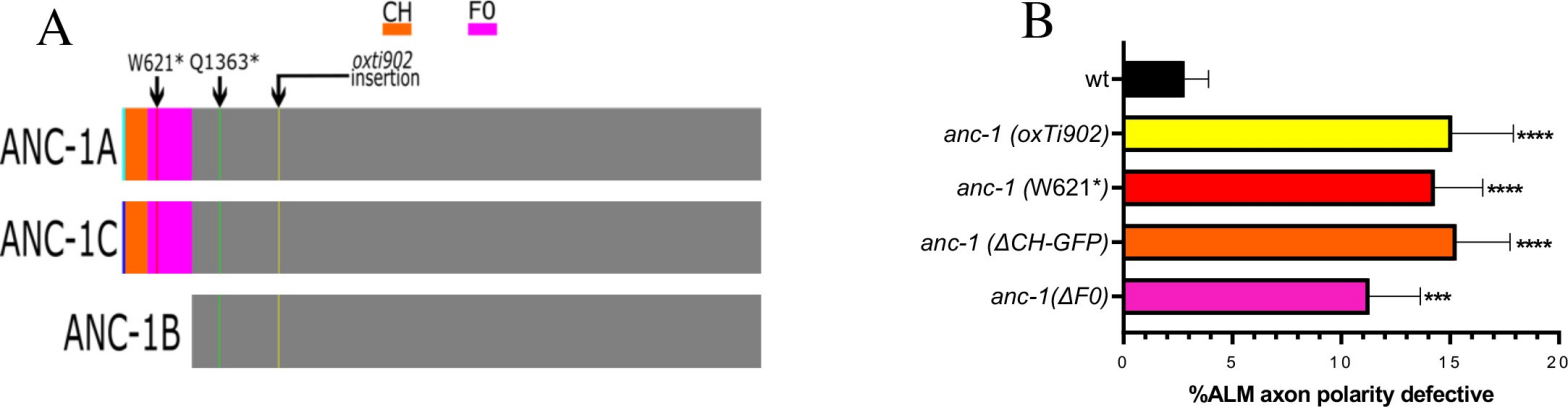
The ANC-1A and ANC-1C isoforms are required for the axon growth-polarizing activity of the *anc-1* gene. **(A)** Diagram of the ANC-1A, ANC-1B and ANC-1C proteins. The W621* mutation removes function of ANC-1A and ANC-1C and the oxTi902 and Q1363* mutations remove function of all three isoforms. **(B)** Quantification of ALM axon polarity defects. The ANC-1A and ANC-1C isoforms are required for the polarization of ALM axon growth. ALM neurons were analyzed in L4 hermaphrodites using the *muIs32* transgene that encodes *Pmec-7*::*gfp*. N>194 for all genotypes. Asterisks indicate statistically significant difference (***p = .007, ****p < .0001) relative to wild type, “N-1” Chi-squared test for proportions. Error bars represent the standard error of the proportion. anc-1(W621*) is anc-1(gk109018[W621*]); anc-1(ΔCH-GFP) is *anc-1(yc41[Δch*::*gfp3xFLAG*::*anc-1]); anc-1(ΔF0)* is *anc-1(syb5659)*.

We used a candidate approach to learn more about the mechanisms that polarize axon outgrowth. We considered ANC-1 as a candidate because its human homolog has been implicated in neurodevelopmental and neurodegenerative disorders and yet little is known about its function in neurons. To disrupt ANC-1 function, we tested the *anc-1(e1873)* null mutation, which we refer to as anc-1(Q1363*) (Fig 2A). We found that the anc-1(Q1363*) mutation causes a multipolar phenotype in the ALM neuron, indicating that ANC-1 is required to polarize ALM axon outgrowth (Fig 1C). To confirm these results, we also used the *anc-1(oxTi902)* null mutation, which is an insertion of an *eft-3*::*gfp* transgene into the 5’ end of *anc-1* that is predicted to disrupt *anc-1* function (Fig 2A) [49]. We found that *anc-1(oxTi902)* also causes a multipolar phenotype in the ALM (Fig 2B). These results reveal a novel function for ANC-1 in promoting the polarity of ALM axon outgrowth.

To further explore the role of ANC-1 in the polarization of axon growth, we asked if it functions in the UNC-6 or SLT-1 signaling pathways. To test for a role of ANC-1 in the SLT-1 signaling pathway, we analyzed *anc-1(oxTi902)*; *slt-1(eh15)* double null mutants (Fig 1C). We found that the penetrance of ALM polarity defects in *anc-1(oxTi902)*; *slt-1(eh15)* double mutants was not significantly different than in the *anc-1(oxTi902)* or the *slt-1(eh15)* single mutants. This observation indicates that *anc-1* and *slt-1* function in a common genetic pathway and is consistent with a function for ANC-1 within the SLT-1 signaling pathway. To test for a role of ANC-1 in the UNC-6 signaling pathway, we analyzed *anc-1(oxTi902)*; *unc-6(ev400)* double null mutants (Fig 1C). We found that the penetrance of ALM polarity defects in *anc-1(oxTi902)*; *unc-6(ev400)* double mutants was significantly greater than the penetrance of ALM polarity defects in the *anc-1(oxTi902)* and *unc-6(ev400)* single mutants. Taken together, these observations support the idea that ANC-1 functions with SLT-1 to polarize ALM axon growth. Moreover, UNC-6 may also contribute to the polarization of ALM axon growth through a mechanism that may be independent of ANC-1.

### The axon growth-polarizing function of ANC-1 is mediated by its ANC-1A and ANC-1C isoforms

**Fig 3:**
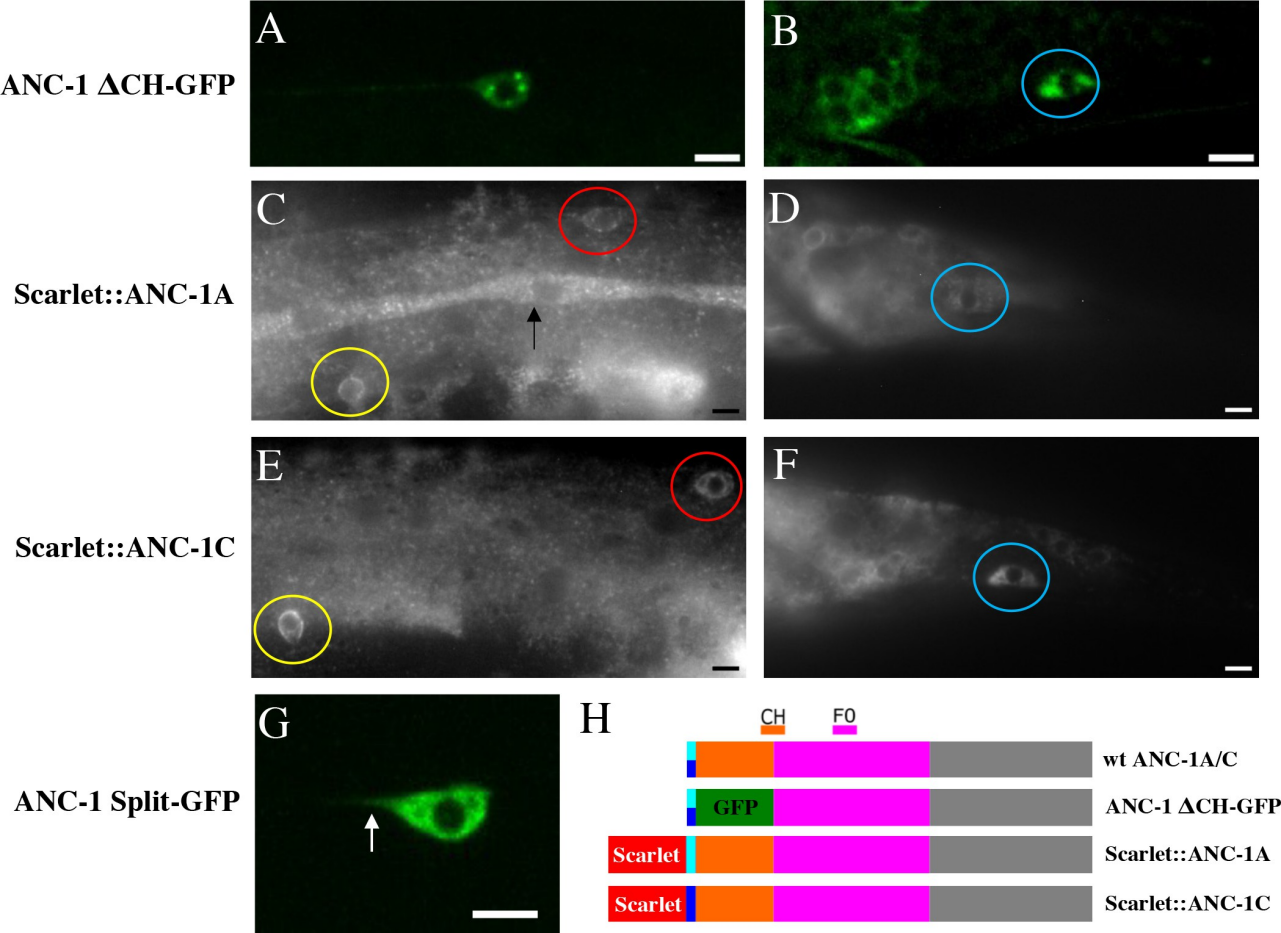
ANC-1A and ANC-1C are enriched in touch receptor neurons and are localized to the base of the proximal axon. **(A)** Expression of ANC-1ΔCH-GFP, a marker for ANC-1A and ANC-1C, in the ALM neuron. **(B)** Expression of ANC-1ΔCH-GFP in the PLM neuron. Also see S1 Fig for colocalization with the Touch Receptor Neuron marker. **(C)** Expression of Scarlet::ANC-1A in the ALM (red circle) and AVM (yellow circle). Expression also seen in the seam cells (see arrow). **(D)** Expression of Scarlet::ANC-1A in the PLM (blue circle). **(E)** Expression of Scarlet::ANC-1C in the ALM (red circle) and the AVM (yellow circle). **(F)** Expression of Scarlet::ANC-1C in the PLM (blue circle). **(G)** ANC-1 visualized by split GFP. The ANC-1 protein was endogenously tagged with 2XGFP11 and GFP1-10 was expressed by a transgene (*Pmec-4*::*gfp1-10)*. **(H)** Schematic of mutant forms of ANC-1. Anterior is to the left. Scales bars are 5 μm.

The *anc-1* gene is expressed as at least three different protein isoforms (Fig 2A). The ANC-1A and ANC-1C isoforms include an N-terminal extension that contains two calponin homology (CH) domains and three spectrin-like repeats. On the other hand, the ANC-1B isoform lacks this N-terminal extension. Previous work has found that the ANC-1B isoform is sufficient to mediate the role of *anc-1* in the anchoring of nuclei in the hypodermis [18]. However, the role of the ANC-1A and ANC-1C isoforms has not been previously demonstrated. We used two mutations to test for a role of ANC-1A and ANC-1C in neuronal polarization. First, we tested the *anc-1(gk109018)* allele, which we refer to as anc-1(W621*). This anc-1(W621*) mutation introduces a stop codon at W621 that causes early termination of the ANC-1A and ANC-1C isoforms, while sparing the ANC-1B isoform (Fig 2A) [18]. We found that the anc-1(W621*) mutation causes ALM polarity defects with a penetrance similar to that of the *anc-1(oxTi902)* null allele (Fig 2B), indicating that the ANC-1A and ANC-1C isoforms are required for the neuron-polarizing activity of the *anc-1* gene. We also tested a second mutation, *anc-1(yc41)*, which we refer to as *anc-1(ΔCH-GFP)* (see Fig 3H). This *anc-1(ΔCH-GFP)* mutation deletes the CH domains of ANC-1A and ANC-1C and replaces them with GFP, while sparing the ANC-1B isoform [18]. We found that *anc-1(ΔCH-GFP)* also causes an ALM multipolar phenotype with a similar penetrance to that observed in the *anc-1* null alleles. Taken together, these observations reveal a role for the ANC-1A and ANC-1C in the polarization of axon growth.

### ANC-1A and ANC-1C are localized to the base of the proximal axon and cell body of the ALM neuron

To investigate the expression pattern of *anc-1a and anc-1c*, we examined GFP expression in *anc-1(yc41)* mutants, which express ANC-1ΔCH-GFP protein, where the CH domains of ANC-1A and ANC-1C are replaced with GFP (Fig 3H). We found that the ANC-1ΔCH-GFP protein is enriched within the touch receptor neurons (ALM, PLM, AVM and PVM) (Figs 3A, 3B, and S1). In addition, we also observed expression within the gonad (Fig 3B). To confirm these findings, we used CRISPR to create two endogenously tagged versions of the *anc-1* gene. The *anc-1(syb5433)* allele expresses N-terminally tagged ANC-1A (Scarlet::ANC-1A) and the *anc-1(syb5416)* expresses N-terminally tagged ANC-1C (Scarlet::ANC-1C). Although these proteins did not express as well as the ANC-1ΔCH-GFP protein, we found that both Scarlet::ANC-1A (Fig 3C and 3D)and Scarlet::ANC-1C (Fig 3E and 3F) are enriched in the touch receptor neurons. We also found expression of Scarlet::ANC1A in the seam cells (Fig 3C). Taken together, these results indicate that expression of ANC-1A and ANC-1C are enriched in touch receptor neurons.

Having found that ANC-1A an ANC-1C are expressed in the touch receptor neurons, we wondered if ANC-1B was also expressed in these neurons. To address this question, we used the *anc-1(yc93)* mutation [18]. This mutation endogenously tags all three isoforms of ANC-1 with GFP, but also introduces stop codons into *anc-1a* and *anc-1c*, thereby allowing for the observation of GFP::ANC-1B expression. We were unable to detect GFP::ANC-1B within the ALM neuron (S2 Fig), suggesting that the ALM expresses only ANC-1A and ANC-1C.

To observe the subcellular localization of ANC-1 specifically within the ALM, we used a split GFP approach [50]. We used CRISPR/Cas9 to create *anc-1(yc112[2xgfp11])* that expresses ANC-1 protein with a 2XGFP11 fragment inserted endogenously in all the isoforms at the N-terminus of ANC-1B and at residue 995 of ANC-1A (ANC-1::2XGFP11). We determined that the *anc-1(yc112[2xgfp11])* allele was functional because it did not cause ALM axon polarity defects that were significantly greater than wildtype controls (4.8±3.3% for yc112 vs. 5.6±1.5% for wt). We then used the *juIs438* transgene, which uses the *mec-4* promoter to drive expression of GFP1-10 specifically within the touch receptor neurons [51], allowing us to visualize the localization of ANC-1::2XGFP11. We found that ANC-1::2XGFP11 was localized to the cell body as well as extending into the base of the proximal axon (Fig 3G).

### Loss of ANC-1A and ANC-1C disrupts the localization of mitochondria in the proximal axon of the ALM

**Fig 4:**
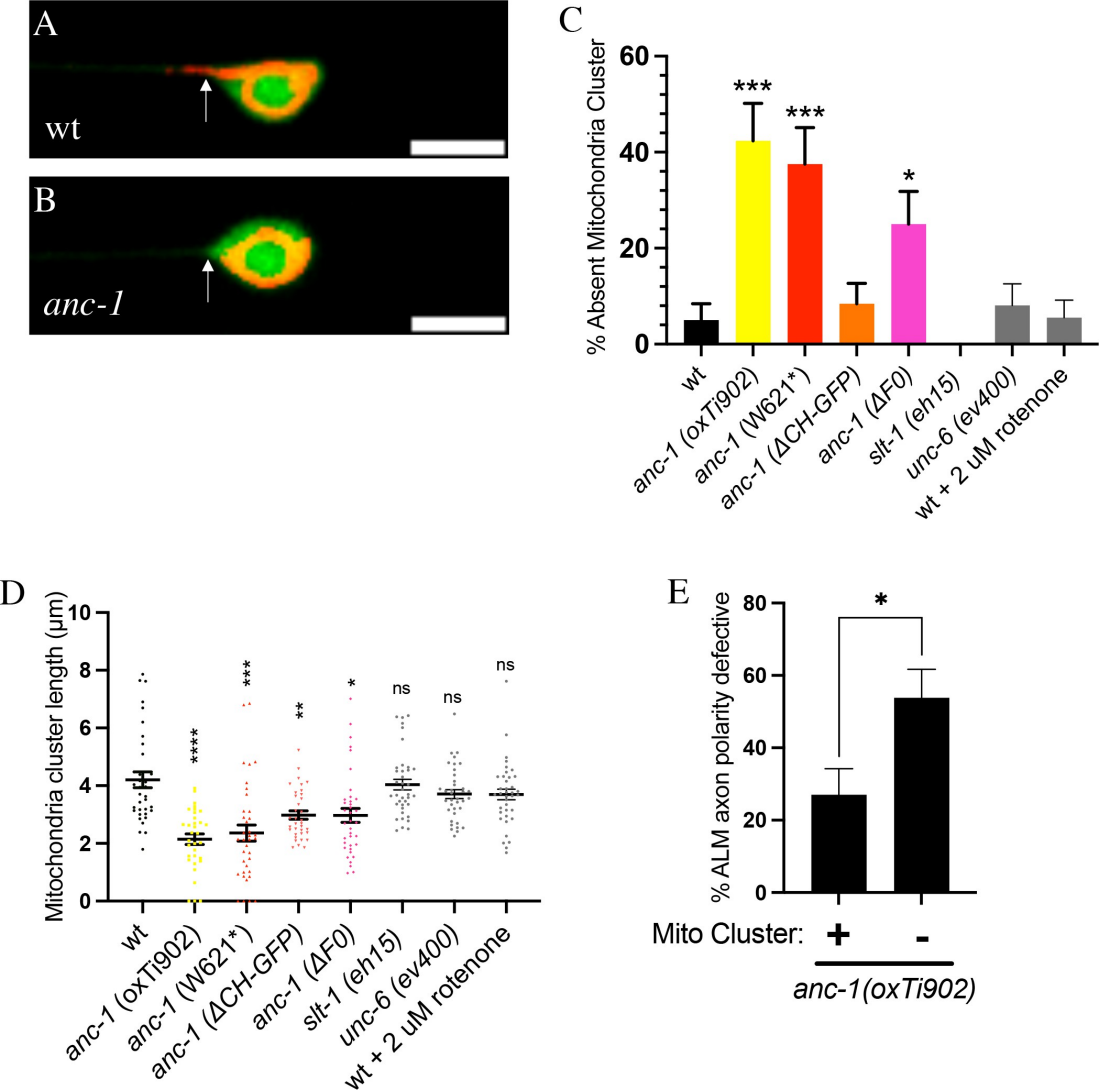
ANC-1A and ANC-1C promote clustering of mitochondria at the base of the proximal axon. **(A-B)** Example of ALM neurons with wildtype shown in (A) and the *anc-1(oxTi902)* null mutant shown in (B). Mitochondria are shown in red and are labeled with the mitochondrial targeting sequence of COX8 fused to TagRFP (MITO::TagRFP), which was expressed from the *jsIs1073* transgene (*Pmec-7*::*mito*::*tagRFP*). The green label is cytosolic GFP, which was used to visualize the entire neuron and was expressed from the *muIs32* transgene *(Pmec-7*::*gfp*). Scale bars are 5 μm. **(C)** Quantification of mitochondrial clustering defect at the base of the proximal ALM axon. N>35 for all genotypes. Asterisks indicate statistically significant difference, “N-1” Chi-squared test for proportions (*p = .016, ***p < .001). Error bars represent the standard error of the proportion. Data displayed as mean ± SEP. **(D)** Quantification of the length of the mitochondria cluster at the base of the proximal axon. N>35 for all genotypes. Data was compared using Welch’s ANOVA and Dunnet’s T3 multiple comparisons tests. Asterisks indicate statistically significant difference, (*p = .011, **p < .01, ***p < .001, ****p < .0001). Data displayed as mean ± SEM **(E)** Correlation between absence of mitochondria at the base of the proximal axon and the ALM axon polarity phenotype. Asterisk indicate statistically significant difference, “N-1” Chi-squared test for proportions (*p = .017). Data displayed as mean ± SEP.

Previous work in cultured mammalian neurons has indicated that mitochondria cluster at the base of the proximal axon and that this cluster is important for axo-dendritic polarity [30,31]. These observations prompted us to ask if *C*. *elegans* ANC-1 could polarize axon growth by controlling the localization of mitochondria within the proximal ALM axon. As a first step in addressing this question, we asked if ANC-1 can regulate the distribution of mitochondria within the proximal ALM axon. In nearly all wild-type ALM neurons, mitochondria were clustered at the base of the proximal axon (Fig 4A and 4C). However, in *anc-1(oxTi902)* null mutants, mitochondria were absent from the base of the proximal axon in 42% of ALM neurons (Fig 4B and 4C), indicating that ANC-1 promotes mitochondria clustering at the base of the proximal axon. Moreover, we also found that the average length of the mitochondrial cluster at the base of the proximal axon was reduced in *anc-1(oxTi902)* relative to wildtype (Fig 4D). Taken together, these results indicate that ANC-1 promotes the correct localization of mitochondria to the base of the proximal ALM axon.

To address the question of the relationship between mitochondria localization defects and the disruption of ALM axon polarity, we looked for a correlation between the mitochondria localization defects and axon polarity defects. We found that *anc-1(oxTi902)* mutant ALM axons that exhibited a defect in mitochondria localization had a penetrance of ALM axon polarity defects that was about two times higher than *anc-1(oxTi902)* mutant ALM axons that did not exhibit mitochondria localization defects (Fig 4E). This observation suggests a correlation between ALM mitochondria localization defects and ALM axon polarity defects.

ANC-1A and ANC-1C each contain an N-terminal extension that is not present in ANC-1B (Fig 2A). This N-terminal extension consists of two CH domains, as well as a region that we call the F0 domain. To determine if the ANC-1A and ANC-1C isoforms are required for localization of mitochondria to the base of the proximal axon, we observed the localization of mitochondria in the anc-1(W261*) mutant. The anc-1(W621*) mutant creates a premature stop codon within the *anc-1a* and *anc-1c* isoforms, but does not alter shorter *anc-1b* isoform. We found that the anc-1(W261*) mutation causes a failure of mitochondria localization to the base of the proximal axon in 38% of ALM neurons, which is not significantly different than the penetrance observed in *anc-1* null mutants (Fig 4C). Moreover, we also found that the average length of the mitochondrial cluster at the base of the proximal axon was reduced in anc-1(W261*) mutants relative to wildtype (Fig 4D). These observations, suggest that the ANC-1A and ANC-1C isoforms are responsible for the clustering of mitochondria at the base of the proximal axon.

To investigate a potential role for the F0 domain in the localization of mitochondria to the base of the proximal axon, we used the *anc-1(syb5659)* mutation. This mutation creates an in-frame deletion in the F0 domain and is hereafter referred to as the *anc-1(ΔF0)* mutation. We found that the *anc-1(ΔF0)* mutation caused a failure of mitochondria localization to the base of the proximal axon in 24% of ALM neurons (Fig 4C). This penetrance is significantly less than we observed for *the anc-1(oxTi902)* null and the anc-1(W621*) alleles. We also found that the *anc-1(ΔF0)* mutation causes a decrease in the length of the mitochondrial cluster at the base of the proximal axon relative to wildtype that is less than the decrease caused by the *anc-1(oxTi902)* null allele and the anc-1(W621*) allele (Fig 4D). Taken together, these observations support the idea that the F0 domain is partially responsible for the ability of ANC-1A and ANC-1C to promote localization of mitochondria to the base of the proximal axon. However, we cannot exclude the possibility that the *anc-1(ΔF0)* mutation reduces overall expression levels of ANC-1A and ANC-1C.

To investigate a potential role for the CH domains in the localization of mitochondria to the base of the proximal axon, we used the *anc-1(ΔCH-GFP)* mutation that deletes the CH domains and replaces them with an in-frame GFP insertion (Fig 3H). We counted the number of ALM axons that exhibited a complete loss of mitochondria localization to the base of the proximal axon and found no significant difference between the *anc-1(ΔCH-GFP)* mutants and wildtype (Fig 4C). However, when we measured the length of the mitochondrial cluster at the base of the proximal axon, we found that the *anc-1*(ΔCH-GFP) mutation caused a decrease relative to wildtype (Fig 4D). Although less than wildtype, the length of the mitochondrial cluster was significantly greater in *anc-1(ΔCH-GFP)* mutants relative to the *anc-1(oxTi902)* null mutants and the anc-1(W621*) mutants. Taken together, these observations support the idea that the CH domains are partially responsible for the role of the ANC-1A and ANC-1C isoforms in promoting the localization of mitochondria to the base of the proximal axon. However, we cannot exclude the possibility that the function of other domains of ANC-1 could be indirectly disrupted by the presence of GFP in the *anc-1(ΔCH-GFP)* mutants.

Since our results indicate that SLT-1 functions in a pathway with ANC-1 to promote the polarization of ALM axon growth, we asked if loss of *slt-1* function would disrupt the localization of mitochondria to the base of the proximal axon. We found that the *slt-1(eh15)* null allele does not alter the localization of mitochondria to the base of the proximal axon (Fig 4C and 4E). Moreover, we found that the *unc-6(ev400)* null allele also does not alter the localization of mitochondria to the base of the proximal axon (Fig 4C and 4E). These observations may reflect the possibility that SLT-1 could affect the activity of mitochondria without affecting their localization. Consistent with this idea, prior studies have indicated that various guidance cues can alter mitochondrial membrane potential without altering mitochondria localization [52,53]. Moreover, we found that treatment with rotenone does not alter localization of mitochondria to the base of the proximal axon (Fig 4C and 4D), suggesting that localization of mitochondria to the base of the proximal axon is independent of mitochondrial membrane potential.

### ANC-1 functions with mitochondria to polarize axon growth

**Fig 5:**
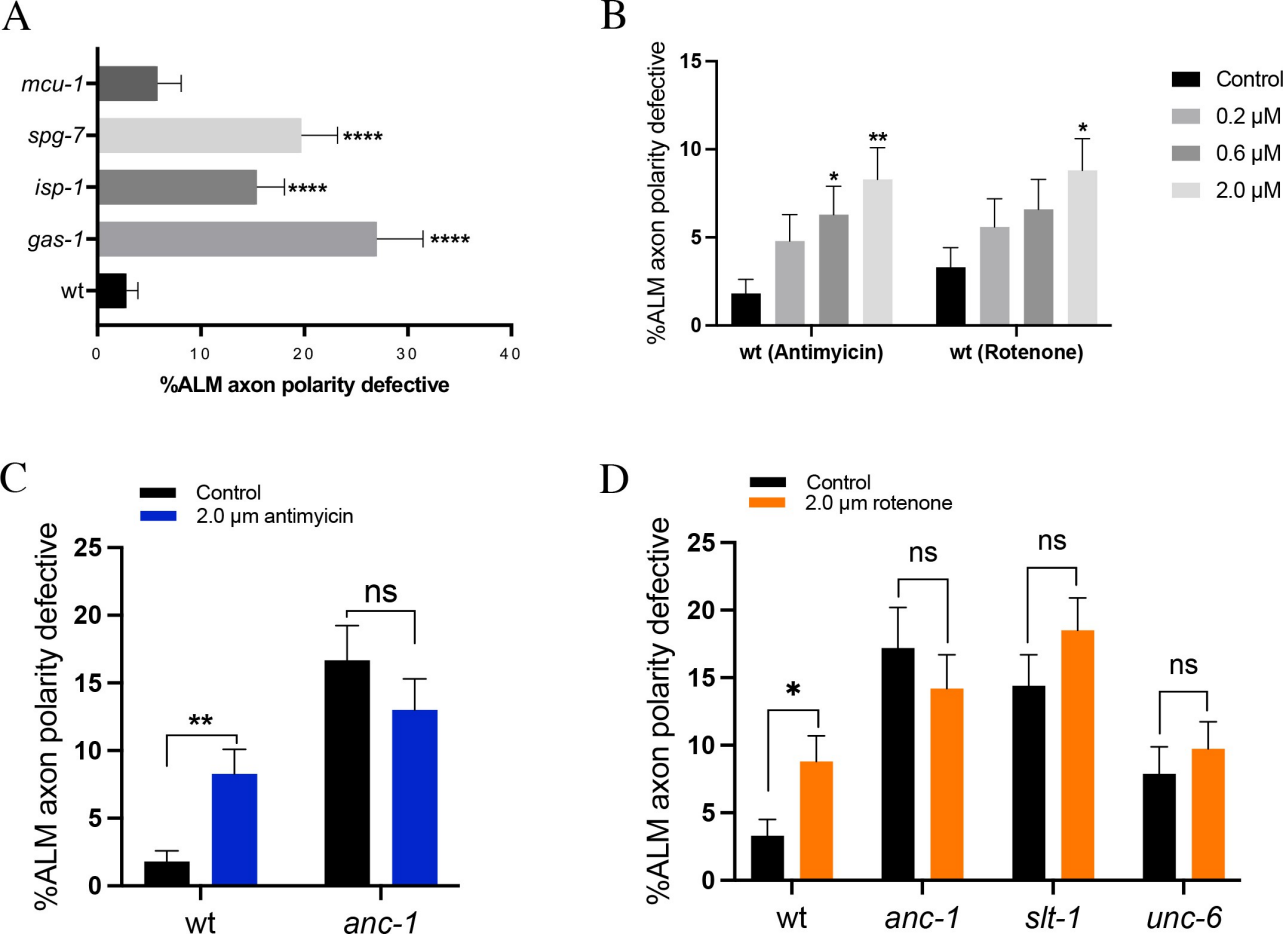
ANC-1 functions with mitochondria to polarize ALM axon growth. **(A)** Mutations that disrupt the electron transport chain cause ALM axon polarity defects. **(B)** Dosage-dependent disruption of ALM axon polarity by Antimycin and Rotenone. **(C)** Antimycin disrupts ALM axon polarity but fails to enhance ALM axon polarity defects caused by the *anc-1(oxTi902)* null mutant. **(D)** Rotenone disrupts ALM axon polarity but fails to enhance ALM axon polarity defects caused by null mutants in *anc-1*, *slt-1*, or *unc-6*. N>196 for each concentration. Asterisks indicate statistically significant difference, “N-1” Chi-squared test for proportions (*p < .05, **p < .01). Error bars represent the standard error of the proportion. Scale bars are 5 μm. Alleles are: *anc-1(oxTi902)*, *mcu-1(ju1154)*, *spg-7(ad2249)*, *isp-1(qm150)*, *gas-1(fc21)*, *unc-6(ev400)*, and *slt-1(eh15)*.

Previous work has indicated that a cluster of mitochondria at the base of the proximal axon can regulate axo-dendritic polarity in mammalian neurons [30,31]. Our finding that ANC-1 is required to polarize axon growth and for clustering of mitochondria to the base of the proximal axon suggested the possibility that ANC-1 could function through mitochondria to polarize axon growth. As a first step in investigating this hypothesis, we asked if mitochondria function is required to polarize ALM axon growth. For this experiment, we used hypomorphic loss of function mutations to partially disrupt the function of three genes that are required for mitochondria function. The *spg-7* gene encodes a AAA metalloprotease that is needed for assembly of the protein complexes that comprise the electron transport chain [54].The *isp-1* gene encodes a component of the electron transport chain complex III and *gas-1* encodes a component of the electron transport chain complex I [55]. We found that reduction of function in any of these three genes cause ALM polarity defects (Fig 5A). These observations suggest that normal mitochondria function is required to polarize ALM axon growth.

Considering that mitochondria function is required for the polarization of ALM axon growth, we next asked if mitochondria function with ANC-1 to polarize ALM axon growth. If so, we would expect that inhibition of mitochondria function would fail to enhance ALM axon polarity defects in the *anc-1* null mutant background. We were unable to obtain double mutants between *anc-1* and *spg-7*, *gas-1* or *isp-1*. As an alternative, we disrupted mitochondrial function by using antimycin to inhibit complex III of the electron transport chain and rotenone to inhibit complex I of the electron transport chain. We found that both antimycin and rotenone induced dose-dependent increases in ALM polarity defects (Fig 5B). However, treatment of *anc-1* null mutants with antimycin failed to enhance ALM axon polarity defects relative to untreated *anc-1* null mutants (Fig 5C). Likewise, treatment of *anc-1* null mutants with rotenone also failed to enhance ALM axon polarity defects relative to untreated *anc-1* null mutants (Fig 5D). Together, these observations suggest that ANC-1 functions with mitochondria to polarize ALM axon growth.

Our results suggest that SLT-1 functions in a pathway with ANC-1 to promote the polarization of ALM axon growth. Moreover, our results also suggest that ANC-1 functions through mitochondria to promote the polarization of ALM axon growth. Therefore, we hypothesized that SLT-1 functions through mitochondria to polarize ALM axon growth. To test this hypothesis, we treated *slt-1(eh15)* null mutants with rotenone. We found that rotenone treatment failed to enhance the penetrance of ALM axon polarity defects in *slt-1(eh15)* null mutants (Fig 5D), supporting the hypothesis that SLT-1 functions through mitochondria to polarize ALM axon growth. In a similar experiment, we also found that rotenone fails to enhance ALM axon polarity defects in *unc-6(ev400)* null mutants.

## Discussion

Mitochondria are important for many aspects of neuronal development and mitochondrial dysfunction has been implicated in neurodevelopmental disorders. Although much has been learned about the mechanisms that regulate mitochondria transport within the axon, little is known about how mitochondria become anchored to particular sites within neurons and how this anchoring orchestrates specific aspects of axonal development. Our work addresses these questions by identifying a mechanism that that causes localization of mitochondria to the base of the proximal axon and promotes polarization of axon growth. Our results indicate that ANC-1 functions in the SLT-1 signaling pathway to promote localization of mitochondria to the base of the proximal axon, and that disruption of ANC-1 function causes ALM polarity defects. Moreover, genetic and pharmacological experiments indicate that ANC-1 functions with mitochondria to polarize ALM axon growth. These results provide insight into how the localization of mitochondria can be regulated to promote neuronal development and suggest a model whereby ANC-1 polarizes axon growth by recruiting mitochondria to the base of the proximal axon.

### A function for ANC-1A and ANC-1C

Our results provide the first identification of a function for ANC-1A and ANC-1C. We report that the ANC-1A and ANC-1C isoforms are required for the polarization of ALM axon growth and for recruitment of mitochondria to the base of the proximal axon. Prior studies have reported that ANC-1A and ANC-1C are dispensable for anchoring of the nuclei in hypodermal cells [18]. Likewise, the function of the N-terminal portion of mammalian Nesprin-1 (encoded by *SYNE1*) has not been identified and is dispensable for the development of epidermis and muscle in mice [56,57]. However, the N-terminal CH domains of Nesprin-2 are required for some types of actin-dependent nuclear migrations [58,59]. A role of Nesprin-2 in neurons is unclear, as Nesprin-2 (encoded by *SYNE2*) is dispensable for neuron migration in rat brain [60]. Moreover, both *SYNE1* and *SYNE2* have been associated with muscular disorders [26]. However, only *SYNE1* has been associated with neurological and neuropsychiatric disorders [10,13].

ANC-1A and ANC-1C contain an N-terminal extension that is not shared with ANC-1B. This N-terminal extension includes two calponin homology domains followed by an uncharacterized region that we call the F0 domain. The F0 domain has not been previously characterized and consists of 676 amino acids. Interestingly, the first 309 amino acids of the F0 domain align with the first three spectrin-like repeats of mammalian Nesprin-1 (43% similarity and 22% identity). Within the spectrin superfamily, CH domains often occur adjacent to spectrin-like repeats [61]. Moreover, structural studies have indicated that the CH domains can function cooperatively with spectrin-like repeats to mediate actin-binding [62]. Therefore, we hypothesize that the CH domains and F0 domains of ANC-1A and ANC-1C function together to promote polarization of ALM axon growth. Future studies will be required to validate and characterize the functions of these domains in this process.

Whereas our results suggest that ANC-1A/C promotes localization of mitochondria to the base of the proximal axon, they do not identify the precise mechanism of how this occurs. One possibility is that the CH domains could function with the F0 domain to promote accumulation of f-actin structures at the base of the proximal axon. Consistent with this idea, prior work has indicated that f-actin is concentrated in the proximal axon of cultured rat cortical neurons [63]. Moreover, other work has indicated that f-actin is important for mitochondrial localization [64].

### Role of mitochondria at the base of the proximal axon

Our findings suggest that the mitochondria at the base of the proximal axon are important for the polarization of axon growth. This idea is supported by our observation that ANC-1 functions with mitochondria to regulate the polarization of axon growth and that the localization of mitochondria to the base of the proximal axon depends on ANC-1 function. Although prior studies have not examined the role of mitochondria in the polarization of axon growth, two studies have implicated mitochondria at the base of the proximal axon in the regulation of axo-dendritic polarity [30,31]. A remaining key question is whether or not polarization is caused by mitochondria at the base of the proximal axon or if other mitochondria elsewhere in the neuron are responsible. A previous study found that selectively disrupting mitochondria at the base of the proximal axon does disrupt axo-dendritic polarity, albeit to a lesser degree than does global disruption of mitochondria [31].

### Mitochondria-independent role of ANC-1 in the polarization of axon growth

In addition to working with mitochondria to promote the polarization of axon growth, our results suggest that ANC-1 may also function independently of mitochondria to polarize axon growth. We report that the *anc-1(ΔCH-GFP)* mutation causes ALM axon polarity defects with a penetrance equal to the *anc-1(null)* alleles. However, we also found that the *anc-1(ΔCH-GFP)* mutation has only a weak effect on mitochondria relative to the *anc-1* null alleles. Taken together, these observations are consistent with the hypothesis that the CH domain of ANC-1 functions both with mitochondria and independently of mitochondria to polarize axon growth. On the other hand, the *anc-1(ΔF0)* mutation causes defects in both axon polarity and mitochondria localization that are similar to those seen in *anc-1(null)* mutants, suggesting that the F0 domain may have a role in axon polarization that is more specific to mitochondria. Future investigations may help to further define the roles of the CH and F0 domains in promoting the polarization of axon growth.

### Role of the SLT-1 and UNC-6 signaling pathways in the polarization of axon growth

Whereas our results suggest that SLT-1, ANC-1 and mitochondria function together to promote axonal polarity, our results are not consistent with a linear model. If SLT-1, ANC-1 and mitochondria function in a linear pathway, we would expect that loss of either SLT-1 or ANC-1 function would disrupt localization of mitochondria at the base of the proximal axon. However, we have found that mitochondria localization is disrupted by loss of ANC-1 function but not by loss of SLT-1 function. Therefore, we propose that SLT-1, ANC-1 and mitochondria function in a non-linear pathway to regulate axonal polarity (see S3 Fig). In this model, ANC-1 regulates the localization of mitochondria to the base of the proximal axon. We hypothesize that SLT-1 does not regulate localization of mitochondria, but rather regulates the function of mitochondria. This model predicts that axonal polarity requires both the normal function and normal localization of mitochondria at the base of the proximal axon and is therefore consistent with our finding that null mutants in *anc-1* and *slt-1* do not enhance each other’s axonal polarity phenotype.

We are unable to directly test the idea that SLT-1 can regulate mitochondria function. However, we found that pharmacological disruption of mitochondria function fails to enhance axon polarity defects in *slt-1* mutants. This observation is consistent with the idea that SLT-1 can promote axonal polarity through the regulation of mitochondria function. In addition, prior studies in other systems have found that other guidance cues can affect mitochondrial membrane potential. For example, semaphorin 3A and CSPGs can regulate mitochondrial membrane potential without affecting mitochondria localization [52,53].

Whereas our work indicates a role for the SLT-1 extracellular cue and its SAX-3 receptor in the polarization of axon growth, more work is needed to fully understand how this guidance cue and receptor promote polarization. For example, we have reported that both SLT-1 and SAX-3 promote axonal polarity. However, we have not examined if loss of SAX-3 function disrupts mitochondria localization. We have also not determined how *sax-3;anc-1* double mutants affect axonal polarity or mitochondrial localization. In this regard, we were unable to obtain these double mutants, possibly due to the strong lethal phenotype of *sax-3*. In fact, prior work has indicated that SLT-1 and SAX-3 do have functions independent of each other [65–68], suggesting the need to further investigate how they interact to control axonal polarization. Thus, future work may expand on our findings to provide a better understanding of how SLT-1 and SAX-3 interact to control axonal polarity.

The role of UNC-6 in the polarization of axon growth will also be an area for future investigation. We have found that a null allele in *unc-6* causes a weak polarity phenotype. Moreover, this *unc-6* null mutation enhances polarity defects in the *anc-1* mutant background, further supporting a role for UNC-6 in the polarization of axon growth. Unexpectedly, we also found that the *unc-6* null mutation does not enhance polarity defects in the *slt-1* null mutant background. This is surprising, because prior work has indicated that UNC-6 and SLT-1 function in parallel to mediate the ventral guidance of the AVM and PVM axons [45,66,69]. Thus, future work will be needed to understand how UNC-6, SLT-1 and their receptors interact to polarize axon growth.

## Materials and methods

### Genetics

*C*. *elegans* strains were maintained according to standard procedures. Strains were cultured on NGM plates seeded with OP50 and kept at 20°C. Standard crosses were performed to obtain some of the strains used in this study and worms were genotyped by PCR and/or sequencing. Alleles used in this study were: *slt-1(eh15)*, *unc-6(ev400)*, *anc-1(oxTi902)*, *anc-1(e1873)*, *anc-1(syb5433)*, *anc-1(syb5416)*, *anc-1(syb5659)*, anc-1(gk109018[W621*]); anc-1(yc41[Δch::*gfp3xFLAG*::*anc-1])*, *mcu-1(ju1154)*, *spg-7(ad2249)*, *isp-1(qm150)*, *gas-1(fc21)*, *anc-1(yc93)*, *and anc-1(yc112)*. Transgenes used in this study were: *cueSi31[Pmec-7*::*CH*:*gfp*::*tbb-2 3’UTR]*, *muIs32 [Pmec-7*::*gfp]*, *and jsIs973[Pmec-7*::*mRFP]*, *jsIs1073[Pmec-7*::*MTS*::*TagRFP]*. MTS is the mitochondrial targeting sequence of COX8. These alleles and transgenes have been previously described with the exception of the following:

The *anc-1(oxTi902)* allele was generated by Christian Frøkjaer-Jensen and obtained from the CGC. This allele is a single copy insertion of *Peft-3*::*gfp* made by miniMos [49]. The *Peft-3*::*gfp* was randomly inserted into the *anc-1* gene, as determined by inverse PCR and sequencing of the flanking DNA sequence [49].

CRISPR alleles were generated by SunyBiotech and include: ***anc-1(syb5433)***: CRISPR/CAS9 was used to insert a sequence encoding 3xFLAG::Scarlet::3xGAS into the *anc-1* gene, just before the start codon for ANC-1A; ***anc-1(syb5416)***: CRISPR/CAS9 was used to insert a sequence encoding 3xFLAG::Scarlet::3xGAS into the *anc-1* gene, just before the start codon for ANC-1C; ***anc-1(syb5659)***: CRISPR/CAS9 was used to create an in-frame deletion that removes the F0 domain (amino acids #524–931 in ANC-1A).

### Phenotype analysis

For analysis of the ALM axon polarity defect, L4 worms were mounted on 5% agarose slides and anesthetized with levamisole for observation with a 40x objective. ALM neurons were scored as having an axon polarity defect if the ectopic process was longer than the length of the cell body. ALM neurons were visualized with the *muIS32* transgene which encodes *Pmec-7*::*gfp*.

For mitochondrial analysis, L4 worms were anaesthetized with levamisole and mounted on 5% agarose slides with a coverslip. Mitochondria were visualized in the proximal ALM using the *jsIs1073* transgene and z stack images were obtained at 60x using Images used for quantitation were acquired using a Nikon Ti inverted microscope equipped with a spinning disk confocal system (Crest Optics) and dual sCMOS cameras (Teledyne Photometrics). Maximum intensity composite images of the mitochondria in proximal ALM axons were assessed for the presence of a mitochondrial cluster in the base of the proximal axon. ALM neurons were binarily scored according to the presence of a narrow strip of mitochondria extending out of the soma into the initial part of the axon. To quantify the length of this extension, the line tool in Fiji was used to measure the distance from the most distal part of the mitochondrial cluster to the boundary of the nuclear space, within the soma, which lacked mitochondrial fluorescence (see S4 Fig).

### Drug treatment

Freshly made stock solutions of rotenone and antimyicin were prepared in DMSO and Ethanol, respectively. The stock solutions were diluted with M9 buffer and vortexed before being spread on plates containing 9 mL of NGM. Final plate concentrations were 2 um rotenone (.08% DMSO) and 2 um antimycin (0.16% ethanol). Control plates received an equivalent amount of the appropriate vehicle diluted in M9. The plates were then wrapped in foil overnight to allow for even diffusion before being coated with with 65 ul of OP50 and being allowed to dry for 24 hours. Four L4 worms were then placed on each of the plates the ALMs of their progeny were screened at the L4 stage for ALM polarity defects.

## Supporting information

S1 Fig ANC-1A and/or ANC-1C are expressed in the ALM and PLM neurons. To visualize expression of ANC-1A/C we used the *anc-1(yc41)* mutation. This mutation deletes the CH domains of ANC-1A and ANC-1C and replaces them with GFP, thereby causing expression of the ANC-1ΔCH-GFP mutant protein in cells that normally express ANC-1A and ANC-1C. We also used a *Pmec-7*::*rfp* transgene to mark touch receptor neurons, including the ALM and PLM. We found that ANC-1ΔCH-GFP is expressed in cells that correspond to touch receptor neurons.
(PDF)

S2 Fig ANC-1B is not expressed in the ALM neuron. To visualize expression of ANC-1B we used the *anc-1(yc93)* mutation. This mutation inserts GFP at the N-terminus of ANC-1B and inserts stop codons into the sequence coding for ANC-1A and ANC-1C, thereby causing expression of GFP::ANC-1B in the absence of ANC-1A and ANC-1C. We also used a *Pmec-7*::*rfp* transgene to mark touch receptor neurons, including the ALM. We were unable to detect ANC-1B in the ALM neuron or in any of the other touch receptor neurons.
(PDF)

S3 Fig Hypothetical model for the roles of SLT-1, ANC-1, and mitochondria in the polarization of axon growth. Our genetic analysis suggest that SLT-1 and ANC-1 function in a pathway to polarize axon growth. Moreover, our pharmacological experiments suggest that both SLT-1 and ANC-1 regulate mitochondria function to promote the polarization of axon growth. However, loss of ANC-1 function disrupts mitochondria localization and loss of SLT-1 function does not disrupt mitochondrial localization. Therefore, we conclude that ANC-1 regulates the localization of mitochondria to the base of the proximal axon (see solid arrow). We also hypothesize that SLT-1 regulates the function of mitochondria without affecting their localization (see dashed arrow). This hypothesis predicts that axonal polarization requires proper function and localization of mitochondria at the base of the proximal axon.
(PDF)

S4 Fig Measurement of the length of the mitochondria cluster at the base of the proximal axon. The upper figure shows an example of the mitochondria cluster in a wildtype ALM neuron. The lower figure shows an example of the mitochondria cluster in an *anc-1(ΔCH-GFP)*. The length of each mitochondria cluster was measured using the line tool in Fiji (see Materials and Methods).
(PDF)

S1 Data Excel file containing the numerical data for Figs 1–5. (XLSX)
