# Supplementary figures and images for: The ANC-1 (Nesprin-1/2) organelle-anchoring protein functions through mitochondria to polarize axon growth in response to SLT-1
Source: PLoS Genet. 2022 Nov 21;18(11):e1010521. doi: 10.1371/journal.pgen.1010521 (PMC9721489; doi:10.1371/journal.pgen.1010521)

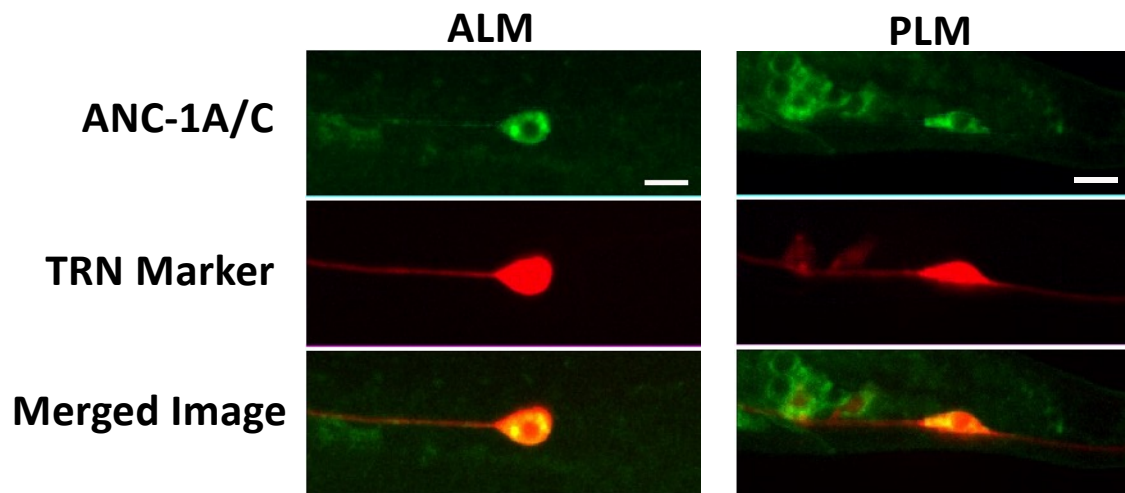

**S1 Figure**

Supplement: S1 Fig — To visualize expression of ANC-1A/C we used the anc-1(yc41) mutation. This mutation deletes the CH domains of ANC-1A and ANC-1C and replaces them with GFP, thereby causing expression of the ANC-1ΔCH-GFP mutant protein in cells that normally express ANC-1A and ANC-1C. We also used a Pmec-7::rfp transgene to mark touch receptor neurons, including the ALM and PLM. We found that ANC-1ΔCH-GFP is expressed in cells that correspond to touch receptor neurons. (PDF) [file pgen.1010521.s001.pdf]

**GFP::**ANC-1B****

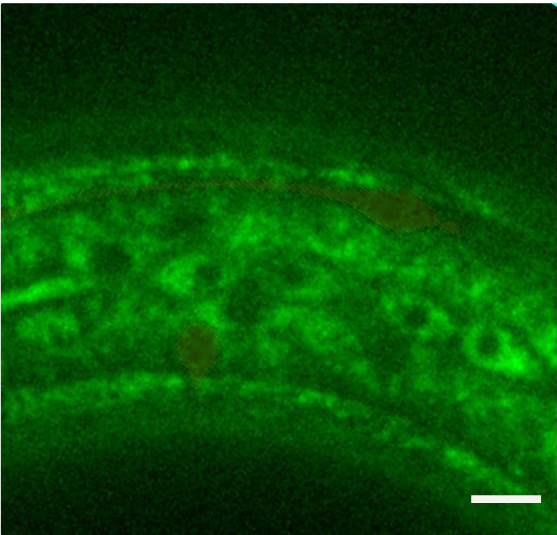

**TRN marker**

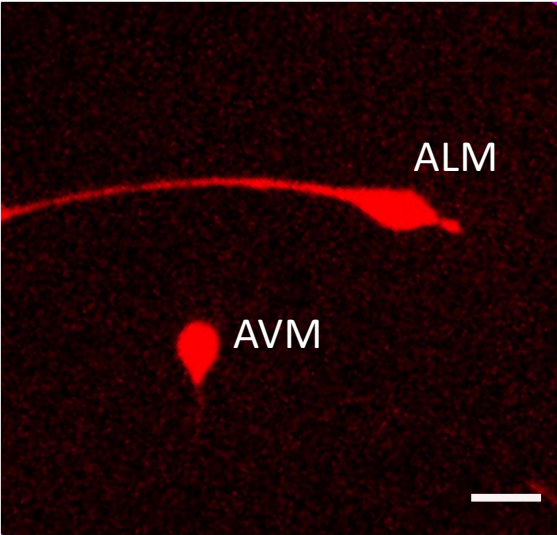

**Merge**

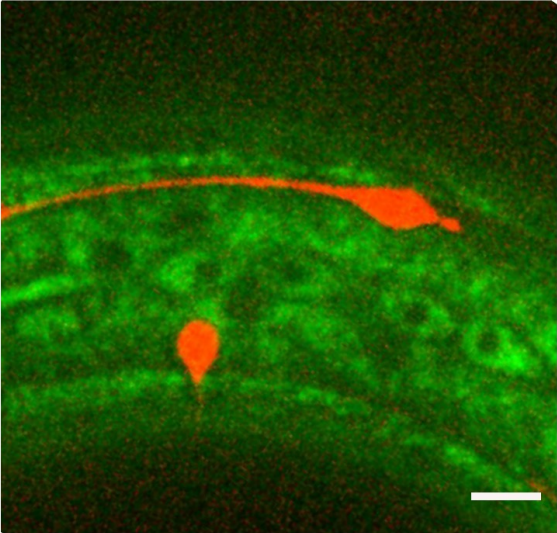

S2 Figure

Supplement: S2 Fig — To visualize expression of ANC-1B we used the anc-1(yc93) mutation. This mutation inserts GFP at the N-terminus of ANC-1B and inserts stop codons into the sequence coding for ANC-1A and ANC-1C, thereby causing expression of GFP::ANC-1B in the absence of ANC-1A and ANC-1C. We also used a Pmec-7::rfp transgene to mark touch receptor neurons, including the ALM. We were unable to detect ANC-1B in the ALM neuron or in any of the other touch receptor neurons. (PDF) [file pgen.1010521.s002.pdf]

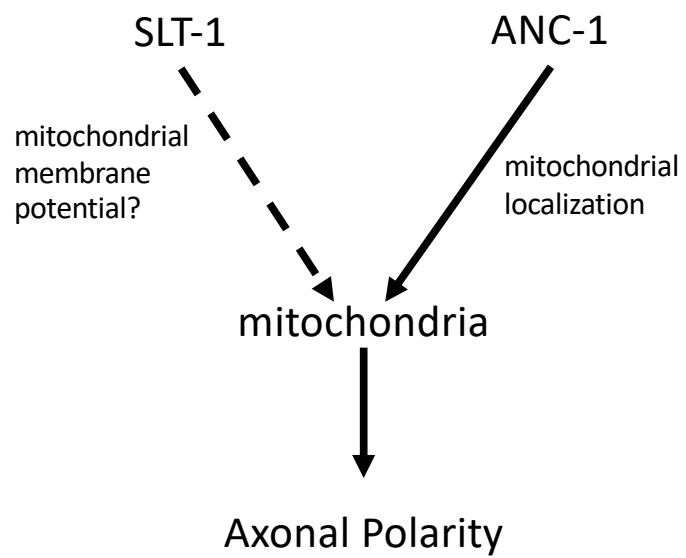

Supplement: S3 Fig — Our genetic analysis suggest that SLT-1 and ANC-1 function in a pathway to polarize axon growth. Moreover, our pharmacological experiments suggest that both SLT-1 and ANC-1 regulate mitochondria function to promote the polarization of axon growth. However, loss of ANC-1 function disrupts mitochondria localization and loss of SLT-1 function does not disrupt mitochondrial localization. Therefore, we conclude that ANC-1 regulates the localization of mitochondria to the base of the proximal axon (see solid arrow). We also hypothesize that SLT-1 regulates the function of mitochondria without affecting their localization (see dashed arrow). This hypothesis predicts that axonal polarization requires proper function and localization of mitochondria at the base of the proximal axon. (PDF) [file pgen.1010521.s003.pdf]

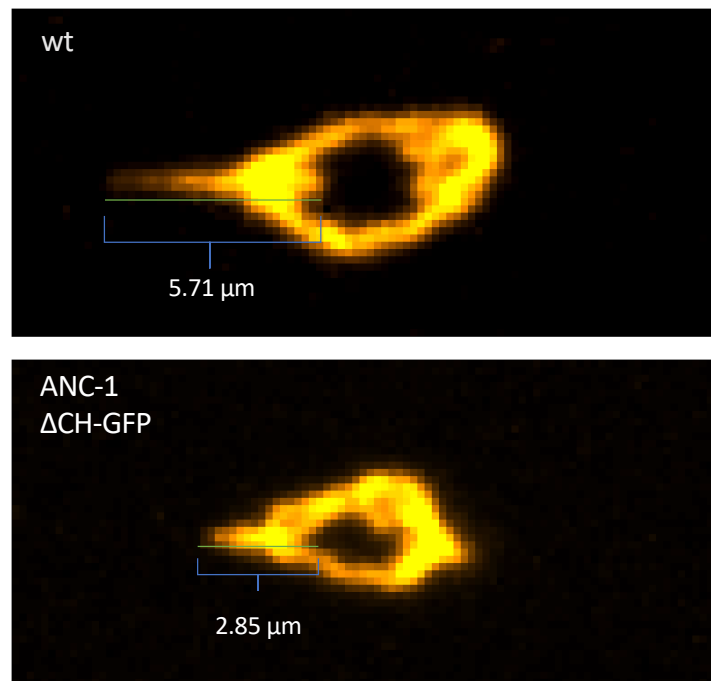

**S4 Figure**

Supplement: S4 Fig — The upper figure shows an example of the mitochondria cluster in a wildtype ALM neuron. The lower figure shows an example of the mitochondria cluster in an anc-1(ΔCH-GFP). The length of each mitochondria cluster was measured using the line tool in Fiji (see Materials and Methods). (PDF) [file pgen.1010521.s004.pdf]
